# The Influence of Interpersonal Sensitivity on Smartphone Addiction: A Moderated Mediation Model

**DOI:** 10.3389/fpsyg.2021.670223

**Published:** 2021-07-22

**Authors:** Li Lin, Xiaochen Wang, Qing Li, Bingnan Xia, Peihong Chen, Wei Wang

**Affiliations:** ^1^School of Business Administration, Zhejiang Gongshang University, Hangzhou, China; ^2^School of Marxism, Zhejiang University of Media and Communications, Hangzhou, China; ^3^Zhongxing Hospital, Hangzhou, China

**Keywords:** interpersonal sensitivity, fear of missing out, smartphone addiction, relational self-construal, college students

## Abstract

Smartphone addiction is a behavioral dependence characterized by excessive or compulsive Internet use and a preoccupation with and loss of control over this use that interferes with an individual’s daily functioning and results in negative mental processes and subsequent social consequences. Smartphone addiction can negatively impact physical and mental health as well as academic performance, sleep quality, and even interpersonal interaction and relationships. Based on the compensatory Internet use theory, this study explores the relationship between interpersonal sensitivity and smartphone addiction in college students and constructed a moderated mediation model. A sample of 881 college students was tested using the Interpersonal Sensitivity Scale, Smart Phone Addiction Scale, Fear of Missing Out Scale, and Relational Self-Construal Scale. We used AMOS 26.0 to conduct a confirmatory factor analysis and employed SPSS 24.0 to test our hypotheses. The results indicated that (1) interpersonal sensitivity was positively related to the fear of missing out and smartphone addiction; (2) the fear of missing out mediated the relationship between interpersonal sensitivity and mobile phone addiction; (3) relational self-construal moderated interpersonal sensitivity and the fear of missing out; and (4) relational self-construal moderated the mediating effect of the fear of missing out on the relationship between interpersonal sensitivity and smartphone addiction. We concluded that the fear of missing out and relational self-construal play a moderated mediation effect on the relationship between smartphone addiction and interpersonal sensitivity. Our findings provided some theoretical implications. Specifically, in addition to proposing a new approach for the study of smartphone addiction, we also introduced a theoretical basis for psychotherapy and intervention of smartphone addiction. In addition, this study also provides some insightful ideas for educational practitioners.

## Introduction

Preoccupied while on his smartphone, a college student in Nanjing, China bumped into a sculpture and knocked it down while walking on campus. A teenager in Zhejiang, China is physically paralyzed and suffers from severe mental deficiency due to excessive use of his mobile phone. Feeling left out and deserted, an elderly man became violent and erratic when his children and grandchildren spent all of their time playing with their mobile phones. The rapid development of mobile Internet, instant messaging, mobile payment, online shopping, and many other mobile applications has made people’s lives more convenient and efficient. However, social problems associated with the overuse of smartphones are increasing as more people become enslaved by their smart phones. Scholars define these uncontrollable mobile phone use behaviors as smart phone addiction, which refers to a new type of addiction that is causing widespread psychological and behavioral problems (Liu et al., 2017). Individual social function is being impaired due to the excessive use of smart phones and the often uncontrollable behaviors that result. Mobile phone addiction seems to be particularly widespread in colleges and universities. Cao (2018) found that the rate of mobile phone addiction among Chinese college students was 25.39%, and 51.81% of college students have experienced decreased efficiency in either work or study due to excessive use of mobile phones. Mobile phone addiction is seriously damaging the physical and mental health of college students, which is affecting their academic performance (Lepp et al., 2014), sleep quality (Yu and Liu, 2019), interpersonal relationship (Chen et al., 2016), etc.

There are several factors that seem to influence college students’ mobile phone addiction. Among these factors, personality trait is a major predictor in studies on addictive behaviors (Dodes, 2009). Previous studies conducted in Chinese context have found that personality factors, such as extroversion, impulsivity, neuroticism, and high risk-taking can positively predict smartphone addiction (Wang et al., 2014). In their research on air force soldiers in China, Wang et al. (2015) found that interpersonal sensitivity is related to neuroticism and negative emotions while negative emotion plays a partial mediating role between neuroticism and interpersonal sensitivity. As a stable and common personality trait, interpersonal sensitivity has not yet been widely considered in studying its interaction with smartphone addiction. Therefore, determining whether or not interpersonal sensitivity affects mobile phone addiction will be one of the primary concerns of this paper.

Interpersonal sensitivity is a personality tendency marked by constantly worrying about negative social evaluation. People with interpersonal sensitivity are more likely to perceive a high degree of social threat, so they always keep vigilant of their own evaluation by other people. They tend to take defensive behavior (such as obedience or inhibition) in advance to avoid such evaluation (Marin and Miller, 2013). According to the compensatory Internet use theory, individuals in less favorable circumstances may choose to alleviate their negative emotions through virtual network activities and may tend to select the network media that meets their needs to compensate for the lack of social communication in real life. Individuals who lack social stimulation in real life are more likely to engage in social activities through the Internet (Kardefelt-Winther, 2014). Many studies have confirmed that the compensatory Internet use theory is not only applicable to Internet addiction, but also can be used to explain mobile phone addiction (Elhai et al., 2018). People with interpersonal sensitivity are more likely to use virtual social media on mobile phones to make up for the lack of social interaction in face to face situations. In addition, interpersonal sensitivity is often accompanied by a sense of incompetence, causing people with interpersonal sensitivity to frequently misunderstand other people’s behaviors. This leads to individual discomfort and ultimately avoidance behaviors and social needs dissatisfaction (Marin and Miller, 2013; Babadi-Akashe et al., 2014). This dissatisfaction encourages individuals to further immerse themselves in various kinds of social media on mobile phones. Eventually such an individual begins pay attention to the dynamics of surrounding people, as well as other people’s comments on their own messages in order to meet their inner needs, which results in even greater dependence on mobile social communication (Ye et al., 2019). You et al. (2019) also found that due to the anonymity and escapism of the Internet, those who cannot establish or maintain a normal offline relationships with others are more likely to interact with others online through the Internet or mobile phones. Therefore, people with interpersonal sensitivity are more likely to become addicted to the use of mobile phones. In view of these findings, this study proposes

*Hypothesis* 1: Interpersonal sensitivity has a significant correlative impact on smartphone addiction.

Although individual interpersonal sensitivity may have an impact on mobile phone addiction, the mediating role between the two also needs to be explored in order to better clarify the internal mechanism of mobile phone addiction. Interpersonal sensitivity can lead individuals to pay too much attention to and set very high standards for their own behaviors. These individuals worry about the negative interpersonal consequences caused by making mistakes, which leads to problems related to anxiety and induces specific behaviors, such as mobile phone addiction (Kumari et al., 2012; Buglass et al., 2016; Baker et al., 2016). This paper argues that the anxiety related to “fear of missing out” embedded in the compensatory Internet use theory may help explain the relationship between interpersonal sensitivity and mobile phone addiction. Fear of missing out is a type of diffused anxiety caused by the individual’s fear of missing out on other people’s interesting experiences (Przybylski et al., 2013; Elhai et al., 2019). In real life, people at risk subconsciously check their social media software due to their underlying anxiety related to the fear of missing out. This phenomenon has evolved from the original individual phenomenon to a widespread social syndrome (Li and Ma, 2019). Interpersonally sensitive individuals have a lack of certain specific psychological controls and cannot carry out effective measures to realize self-regulation. Instead, these individuals rely on external material and environmental stimuli to compensate for their psychological need. Continual and chronic excessive attention to external information gradually evolves into the fear of missing out. Empirical studies also show that fear of missing out leads to social media dependence (Oberst et al., 2017), and individuals who are afraid of missing out are more likely to use mobile social media frequently and excessively focus on their friends’ plans and arrangements (Przybylski et al., 2013). A survey study also found that individuals who have the fear of missing out are more likely to use Facebook in class or check information on mobile phones while driving (Rozgonjuk et al., 2019). People who are afraid of missing out will pay more attention to the emotional state of others in social interaction and have a higher demand for recognition, which often leads to overuse of social media, such as smart phones (Oberst et al., 2017). Therefore, this study proposes

*Hypothesis* 2: The fear of missing out mediates the relationship between interpersonal sensitivity and mobile phone addiction.

This study also explores the boundary conditions required in order for interpersonal sensitivity to effectively cause mobile phone addiction, because this impact process is also affected by factors, such as individual self-concept (Zhang and Kang, 2016) and growth experience (Hall et al., 2009). Researchers have found that an individuals’ self-construal can affect his or her emotions and behaviors in social media use (Chang, 2015). Relational self-construal is a specific classification of self-construal wherein individuals construct themselves according to their relationship with other people deemed more important than themselves (Li et al., 2014). Specifically, individuals with a high level of relational self-construal tend to overly consider other people’s feelings and pay excessive attention to relationship maintenance (Nazir and Maya, 2019). They are more sensitive to interpersonal relationships, and they are eager to avoid less favorable evaluation, striving instead for group praise, and acceptance (Liu and Gu, 2015). These individuals believe that missing key information may lead to exclusion from the group. Therefore, in interpersonal communication, these individuals will often pay continuous attention to the latest status of others as well as their own evaluation, and for them, the discomfort caused by missing information is intensified. It can be seen that interpersonal sensitivity has a stronger effect on the fear of missing out for people with higher levels of relational self-construal. On the other hand, individuals with higher levels of relational self-construal are relatively independent and tend to consider their own interests rather than focusing too much on others. They are more likely to take the initiative to avoid spurious interpersonal communication in an attempt to protect themselves from negative evaluation. They do not feel compelled to dwell on other people’s emotions and dynamics simply to integrate into the group, and the anxiety and fear caused by missing information are significantly reduced. It can be seen that the effect of interpersonal sensitivity on the fear of missing out may be weakened for people with lower levels of relational self-construal. This study, then, proposes

*Hypothesis* 3: Relational self-construal may play a key role in the relationship between interpersonal sensitivity and the fear of missing out. When an individual in question is a person with a high level of relational self-construal, the influence of interpersonal sensitivity on the fear of missing out is enhanced.

Hypotheses 2 and 3 further support the model with regard to a moderated mediation effect. For individuals with a high level of relational self-construal, interpersonal sensitivity will lead to anxiety due to the fear of missing out on the latest news and messages in mobile media. This anxiety then leads to mobile phone addiction. For individuals with a low level of relational self-construal, interpersonal sensitivity has less of an effect on the fear of missing out, and thus, it is less likely to induce mobile phone addiction. As Nazir and Maya (2019) have shown, individuals with high levels of independent self-construal are more individualistic. They distance themselves from others so they are less dependent on social media, so for them, the risk of mobile phone addiction is lower. This study, therefore, proposes

**Figure 1:**
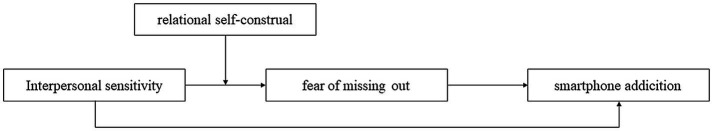
Research model.

*Hypothesis* 4: Relational self-construal moderates the mediating effect of the fear of missing out between interpersonal sensitivity and mobile phone addiction. This means that the higher the level of relational self-construal, the stronger the mediating effect of the fear of missing out. By contrast, the lower the level of relational self-construal, the weaker the mediating effect. The research model is shown in Figure 1.

## Materials and Methods

### Sample

This study randomly selected students as the research sample from six universities in Hangzhou, China, and distributed 881 questionnaires. To avoid the influence of smart-phone usage on the process and results of our data collection, we chose to distribute only paper-based questionnaires rather than online questionnaires. All the questionnaires were distributed and collected with our research team consisting of one professor, one lecturer, one PhD student, and five graduate students. In order to avoid homologous errors and improve data reliability, questionnaires were sent out at two different points in time. We identify the two distribution times as T1 and T2, and there was an interval of 2 weeks between the two distributions. At T1, students were required to answer questions relating to interpersonal sensitivity, relational self-construal, and the fear of missing out. Two weeks later, at T2, the same group of students was required to answer questions relating to smartphone use and addiction. After eliminating invalid questionnaires, 863 valid questionnaires were obtained representing an effective rate of 97.95%. Participants in this survey ranged from freshmen to seniors with an age range between 17 and 23. The average age of participants was 20.93 years old. This study involved 510 female students and 353 male students, and our sample included 591 liberal arts students, 242 science and engineering students, and 30 art students. In addition, since this study was carried out in China with Chinese students involved, hence, all materials, including all scales were adapted to Chinese language.

### Data Collection Instruments

This study used a five-point Likert-type scale, with responses ranging from “not applicable,” coded as 1, to “always,” coded as 5.

#### The Interpersonal Sensitivity Measure

The 36-item Interpersonal Sensitivity Measure (IPSM) developed by Boyce and Parker (1989) was used, which included five structural dimensions: “interpersonal awareness,” “needs for approval,” “separation anxiety,” “timidity,” and “fragile inner-self.” The higher the total score, the stronger the interpersonal sensitivity is for an individual. In this study, the Cronbach α coefficient of this scale is 0.86, and the α coefficients of the five dimensions range from 0.61 to 0.73.

The revision process of the Chinese version of IPSM is as follows. First, two university teachers majoring in psychology independently translated the scale into Chinese, and the first draft of the scale is formed through repeated discussion and modification. Then, two university teachers majoring in English were invited to translate and revise the Chinese version of the scale. Finally, each item of the scale was discussed by graduate students majoring in psychology to ensure that the wording of each item conforms to the rules of expression in Chinese.

#### The Smartphone Addiction Scale for College Students

The 22-item smartphone addiction scale for college students (SAS-C) developed by Su et al. (2014) was used, including six structural dimensions: “withdrawal behavior,” “salience behavior,” “social comfort,” “negative effects,” “use of application (app),” and “renewal of app.” According to the criteria proposed by Cao (2018), the individual is regarded as non-addictive to smartphone use when the total score is less than or equal to 65 and is considered as addictive to smartphone use when the total score is more than or equal to 66. The higher the individual total score, the higher the level of smartphone addiction. In this study, the Cronbach α coefficient of the scale is 0.87, and the α coefficients of the six dimensions range from 0.62 to 0.83.

#### The Fear of Missing Out Scale

This study used the 10-item Fear of Missing Out Scale developed by Przybylski et al. (2013). With this scale, the higher the score, the higher the level of fear of missing out. In order to adapt this scale to the specific scenario in China, we drew on the previous studies (Ma and Liu, 2019) and set the background of fear of missing out as “WeChat, QQ space, or Microblog (the three most popular social media most frequently used by youngsters in China).” We used question items, such as “when I had a good time, it is important for me to share details through social networking sites (such as circle of friends in WeChat, QQ space, or Microblog).” In this study, the Cronbach α coefficient of the scale was 0.79.

#### The Relational Self-Construal Scale

The 9-item relational self-construal scale revised by Huang (2012) was used to measure and identify the degree of self-definition according to intimate others. The higher the score, the higher the level of relational self-construal. This scale was originally developed by Cross et al. (2000). After revision, questions eight and nine with a correlation of less than 0.40 in Chinese context were removed. The scale has good reliability and validity in existing studies and is suitable for research in the field of pedagogy and Psychology (Li et al., 2018a). In this study, the Cronbach α coefficient of the scale was 0.83.

### Testing Procedure and Data Processing

In this study, trained students majoring in psychology were selected to conduct the survey, using a unified procedure for group testing with questionnaires collected on the spot. SPSS 24.0 and Amos 26.0 were used for data analysis and testing. Meanwhile, the Bootstrap method *via* the SPSS macro program PROCESS V3.3 was applied to test the mediating effect, so as to test the significance level of the intermediary path within the 95% confidence interval. Also included in the statistical analysis as control variables are the gender, average time of using smartphone, grade, major, only-child in the family or not, parenting style (spoiling type, indulgent type, or democratic type), and growth environment (main caregivers in the growth process: parents, grandparents, or other relatives; attending boarding school or with no caregiver around). Respondents were required to choose one of the types of each control variable; for example, they were asked to select one of the parenting styles from the choices of “spoiling type, indulgent type, and democratic type” in the questionnaires.

## Data Analysis and Results

### Confirmatory Factor Analysis

The Harman single-factor test was used to test common method deviation (Podsakoff et al., 2003). The results revealed the mutation rate interpretation of the first factor was 16.68%, which was less than the critical value of 40%, indicating that there was no obvious deviation of the common method in this study.

We compared our hypothesized model (i.e., model 4, the baseline four-factor model) with a three-factor models (i.e., model 3, combining interpersonal sensitivity and fear of missing out), a two-factor model (i.e., model 2 combining interpersonal sensitivity and fear of missing out and combining smartphone addiction and relational self-construction), and a one-factor model combining all items (i.e., model 1; Table 1). Considering the changes in chi-square (i.e., *χ*^2^), two major fit indicators [i.e., comparative fit index (CFI) and incremental fit index (IFI)], and root mean square error of approximation (RMSEA), our hypothesized four-factor model (with *χ*^2^*/df* =1.79, IFI = 0.91, CFI = 0.91, and RMSEA = 0.03) showed better fit than other alternative models (Bentler and Bonett, 1980; Bagozzi et al., 1991). Therefore, the discriminant validity of the constructs was confirmed. This suggests that the participants of our survey could distinguish the focal constructs clearly.

**Table 1:** Results of confirmatory factor analysis of the measurement models.

Measurement models	*χ*^2^	*df*	*χ*^2^/*df*	RMSEA	IFI	CFI
Model 1: One-factor (combined all items into one factor)	6466.80	2,536	2.55	0.04	0.82	0.82
Model 2: Two-factor (combined IS and FMO into one factor and combined SA and RSC into one factor)	5957.25	2,535	2.35	0.04	0.84	0.84
Model 3: Three-factor (combined IS and FMO into one factor)	5015.34	2,533	1.98	0.03	0.89	0.89
Model 4: Four-factor	4528.70	2,530	1.79	0.03	0.91	0.91

IS, interpersonal sensitivity; FMO, fear of missing out; SA, smartphone addiction; and RSC, relational self-construction.

### Descriptive Statistics and Correlation Analysis

The mean value, standard deviations, Cronbach’s alpha, and correlation coefficient of the variables are shown in Table 2. Correlation analysis showed that interpersonal sensitivity is significantly positively correlated with smartphone addiction (*r =* 0.49, *p* < 0.01) and fear of missing out (*r =* 0.56, *p* < 0.01), and fear of missing out is significantly positively correlated with smartphone addiction (*r =* 0.45, *p* < 0.01). Thus, these results preliminarily support the subsequent regression analysis.

**Table 2:** Descriptive statistics and correlation analysis.

S. No.	Variable	*M*	*SD*	1	2	3	4	5	6	7	8	9	10	11
1.	Gender	1.71	0.46											
2.	Time	3.15	0.71	0.06										
3.	Grade	1.94	1.12	−0.03	0.09^**^									
4.	Major	1.35	0.54	−0.20^**^	−0.01	0.08^*^								
5.	If the one-child	1.53	0.50	0.08^*^	0.06	−0.13^**^	−0.02							
6.	Parenting style	2.80	0.67	−0.01	0.12^*^	0.01	−0.03	−0.04						
7.	Growth environment	1.14	0.44	−0.11^**^	0.00	0.02	0.07	0.15^**^	−0.18^**^					
8.	Smartphone addiction	2.85	0.55	0.02	0.26^**^	0.07^*^	−0.08^*^	0.01	−0.02	−0.02	(0.87)			
9.	Interpersonal sensitivity	3.21	0.39	0.04	0.07	−0.01	−0.07	0.01	−0.05	0.02	0.49^**^	(0.86)		
10.	Fear of missing out	2.97	0.59	0.06	0.09^**^	−0.01	−0.08^*^	0.00	0.00	−0.05	0.45^**^	0.56^**^	(0.79)	
11.	Relational self-construction	3.64	0.53	0.08^*^	0.02	0.03	−0.07^*^	0.01	−0.02	−0.02	0.14^**^	0.39^**^	0.31^**^	(0.83)

* *p* < 0.05;

** *p* < 0.01.

*M*, mean; *SD*, standard deviation.

### Hypotheses Testing

Model 4 (a simple mediation model) in the SPSS expansion macro PROCESS prepared by Hayes (2012, unpublished) was used to test the mediation effect of fear of missing out on the relationship between interpersonal sensitivity and smartphone addiction. Interpersonal sensitivity was a significant predictor of smartphone addiction [*β =* 0.48, SE=0.03, *p <* 0.001, (CI) = (0.40, 0.58)], thus supporting Hypothesis 1. Results of the bootstrapping test [*β* = 0.24, *SE* = 0.02, *p <* 0.001, (CI) = (0.13, 0.25)] supported that CI did not contain zero. Therefore, fear of missing out plays a partial mediating role in the relationship between interpersonal sensitivity and smartphone addiction. The direct (0.49) and mediated (0.19) prediction effects accounted for 72.56 and 27.94% of the overall effect, respectively. These results lend support to our Hypothesis 2.

In the second step, we employed Model 7 in the SPSS extension macro, and the moderated mediation model was tested. As shown in Table 3, after inputting relational self-construction into the model, the interaction between interpersonal sensitivity and relational self-construction was a significant predictor of fear of missing out (Interpersonal sensitivity × Relational self-construction: *β* = 0.04, *SE* = 0.02, *p* < 0.01), indicating that relational self-construction moderated the relationship between interpersonal sensitivity and fear of missing out (Model 1).

**Table 3:** Moderated mediation effect analysis.

	Model 1 (criterion: fear of missing out)				Model 2 (criterion: smartphone addiction)			
	*β*	*SE*	*p*	95% CI	*β*	*SE*	*p*	95% CI
**Control variables**								
Gender	0.03	0.06	0.61	[−0.09, 0.16]	−0.07	0.06	0.29	[−0.19, 0.06]
Time	0.08^*^	0.04	0.05	[0.00, 0.16]	0.30^**^	0.04	0.00	[0.22, 0.38]
Grade	−0.02	0.03	0.42	[−0.07, 0.16]	0.05^*^	0.03	0.04	[0.00, 0.10]
Major	−0.05	0.05	0.36	[−0.15, 0.06]	−0.07	0.05	0.17	[−0.18, 0.03]
If the one-child	−0.02	0.06	0.75	[−0.13, 0.09]	0.02	0.06	0.72	[−0.10, 0.13]
Parenting style	0.05	0.04	0.26	[−0.04, 0.13]	0.03	0.04	0.41	[−0.04, 0.12]
Growth environment	−0.09	0.07	0.17	[−0.22, 0.04]	−0.02	0.07	0.71	[−0.15, 0.10]
**Independent variable**								
Interpersonal sensitivity	0.51^**^	0.03	0.00	[45, 0.57]	0.35^**^		0.00	[0.28, 0.41]
**Mediator**								
Fear of missing out					0.24^**^		0.00	[0.17, 0.30]
**Moderator**								
Relational self-construction	0.13^**^	0.03	0.00	[06, 0.18]				
**Interaction term**								
Interpersonal sensitivity × Relational self-construction	0.04^*^	0.02	0.03	[01, 0.08]				
*R*^2^	0.33				0.34			
*F*	42.76^**^				48.63^**^			

* *p* < 0.05;

** *p* < 0.01.

**Figure 2:**
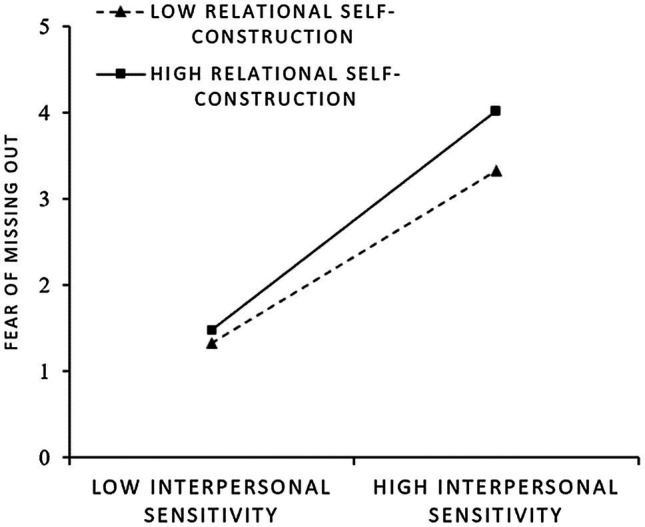
Schematic diagram of interaction effect (fear of missing out).

In addition, we plotted the interaction effects at different levels (i.e., +1 *SD* or −1 *SD*) of relational self-construction using the recommendation of Aiken and West (1991). Figure 2 shows that interpersonal sensitivity is more positively related to fear of missing out when relational self-construction is high rather than low. Accordingly, the moderating effect of relational self-construction on the interpersonal sensitivity–fear of missing out association is as expected, and thus, Hypothesis 3 is supported.

We further estimated the conditional indirect effect of interpersonal sensitivity on smartphone addiction *via* fear of missing out across levels of relational self-construction by bootstrapping the bias-corrected CI. The results are presented in Table 4. The indirect effect of interpersonal sensitivity on smartphone addiction through fear of missing out was stronger and significant at a high level of relational self-construction (effect size = 0.19, 95% bias-corrected CI from 0.13 to 0.25) but was weaker at a low level of relational self-construction (effect size = 0.16, 95% bias-corrected CI from 0.10 to 0.21). Thus, we have further evidence to support our Hypothesis 4.

**Table 4:** Results for conditional indirect effect across levels of relational self-construction.

Level	Effect size	Boot *SE*	LL 95% CI	UL 95% CI
*M−SD*	0.16	0.03	0.10	0.21
*M*	0.17	0.03	0.12	0.23
*M+SD*	0.19	0.03	0.13	0.25

## Discussion

First of all, the results show that interpersonal sensitivity has a significant and positive impact on smartphone addiction. This is because interpersonally sensitive people overreact to negative evaluation and social rejection and will take defensive and remedial measures quickly once they feel alienated from their surrounding interpersonal relationships (Marin and Miller, 2013). For example, WeChat, as the most widely used social media application in China, can help people with interpersonal sensitivity follow the latest activities and status of people around them as well as the comments made on their own WeChat updates. In addition, due to the portability, anonymity, and vast choice of applications on smartphones, interpersonally sensitive individuals will make up for the dissatisfaction in their real social life by using various social media applications on smartphones. This also further supports the previous research results, namely, that individuals with interpersonal sensitivity are more likely to have a variety of addictive behaviors (Taymur et al., 2016; You et al., 2019).

Secondly, based on the compensatory Internet use theory, this study confirms that the fear of missing out plays a partial mediating role between interpersonal sensitivity and smartphone addiction and clarifies the internal influence mechanism of interpersonal sensitivity on smartphone addiction. This is consistent with the findings of the previous studies which suggest that interpersonal sensitivity can lead to social anxiety (Kumari et al., 2012), and the level of anxiety related to the fear of missing out is positively correlated with social media dependence (Oberst et al., 2017). Because of the constant attention paid to the dynamic of other people and the surroundings, individuals with interpersonal sensitivity are prone to the fear of missing new updates, which can be understood as a kind of generalized anxiety. In addition, it is this kind of anxiety that further aggravates their dependence on smartphone use, which leads to smartphone addiction. However, the fear of missing out only partially mediates the relationship between interpersonal sensitivity and smartphone addiction, which means that additional interference may be present. Therefore, future research can further explore the potential influence mechanism on the basis of this study.

Furthermore, this study also found that relational self-construal positively moderates the relationship between interpersonal sensitivity and the fear of missing out. This is because individuals with high levels of relational self-construal define themselves according to specific intimate relationships which lead to changes in self-related cognition (Chang, 2015). Individuals with high levels of relational self-construal pay excessive attention to the feelings of the relatives and friends around them and tend to overtly consider and be affected by the ideas of others. Expecting to form good interpersonal relationships around them, they constantly pay attention to the dynamics of their friends, the fear of missing out on any information about their friends, and worrying about the negative comments of others (Li et al., 2018b). Under the moderation of a high level of relational self-construal, individuals with interpersonal sensitivity will pay close attention to the emotional changes, comments, and activities of the people around them, so they have a higher level of fear of missing out. On the other hand, if the interpersonally sensitive individual holds a lower level of relational self-construal, his or her self-concept will be more stable, so he or she tends to be immersed in his or her own world and has less consideration toward the interaction with others. In such a case, he or she will not have a higher level of fear of missing out and may choose social avoidance to keep away from the emotional changes brought about by interpersonal sensitivity. This avoidance behavior serves to relieve his or her own state of tension.

Finally, the results show that relational self-construal positively moderates the mediating role of the fear of missing out between interpersonal sensitivity and smartphone addiction, a conclusion supported by testing the model. Specifically, compared with individuals with a lower level of relational self-construal, those with a higher level of relational self-construal are more worried about being negatively evaluated by others and more afraid of missing the latest news of others and are therefore more prone to smartphone addiction. This results also support the research findings of the previous studies (Cetin et al., 2012).

From the perspective of interpersonal sensitivity and the fear of missing out, this study explored the influence of these two variables on smartphone addiction. The results not only provide a new research angle for future case analysis of smartphone addiction, but also offer a theoretical basis for the practice of psychotherapy and intervention of smartphone addiction. In recent years, practitioners have begun to pay close attention to the problem of smartphone addiction and actively explore corresponding solutions. For example, the South Korean government has set up special training camps for smartphone addicts, prohibiting addicts from using smartphones for a period of 12 days. The mayor of Bandung city in Indonesia initiated a program in which students were given baby chicks and were then trained to raise them in an attempt to redirect their interest and focus and reduce their dependence on electronic devices. In subsequent procedures relating to clinical intervention, therapists can first solve the problem of interpersonal sensitivity of addicted individuals or alleviate their fear of missing out, and then treat their addictive behaviors accordingly. In practice, colleges and universities should further improve their psychological counseling systems, establish educational programs to correct key cognitive beliefs, and address emotional and behavioral reactions of students with smartphone addiction in interpersonal situations. This will help to reduce the investment of psychological resources into their interpersonal relationships. For students with relatively higher levels of interpersonal sensitivity, this study suggests that intervention through games, group discussions, role play, and other measures may be beneficial. Psychological intervention measures, such as the method of mindfulness, cognitive therapy, and aversion therapy, can be applied to make college students realize the harm of smartphone addiction and then produce aversive reactions to smartphone addiction, so as to reduce or eliminate the behaviors or state related to smartphone addiction (Zhu, 2014).

While there may be some shortcomings in this study, we believe the results are significant and provide a solid basis upon which future research can be based. The limitation may due to the fact that this study only involved college students in Hangzhou, China in the research sample, which could impact the universality of the survey. Future research should consider a much wider geographic sampling area. Secondly, although this study has partially revised the questionnaire related to the fear of missing out and interpersonal sensitivity, the localization of these two concepts still needs to be further explored in future research. Lastly, this study only used methods involving questionnaire-based surveys with self-report scales, while further experimental research is expected to be conducted through more comprehensive data sampling and collection across longer periods of time and employing more sampling events. Expanding this research will provide a better understanding of the dynamics, causations, and correlations relating to the causal relationships and mediating mechanisms between variables.

## Data Availability Statement

The raw data supporting the conclusions of this article will be made available by the authors, without undue reservation.

## Ethics Statement

Ethical review and approval were not required for the study on human participants in accordance with the local legislation and institutional requirements. The patients/participants provided their written informed consent to participate in this study.

## Author Contributions

LL is responsible for the overall development of this study, including the selection of research angle, research dimensions, the planning of sample collection, data analysis and proofreading, and polishing of the whole paper. XW is in charge of the formulation of the general research topic, the construction of the research framework, and the proposing of the theoretical hypothesis. QL generally contributes to the construction of the theoretical framework based on the in-depth accumulation of a large volume of the literature reading and analysis. BX and PC are in charge of data collection and analysis of this study. WW is responsible for all the procedures taken during data collection. All authors contributed to the article and approved the submitted version.

### Conflict of Interest

The authors declare that the research was conducted in the absence of any commercial or financial relationships that could be construed as a potential conflict of interest.
